# 2549. Pharmacometric Analyses to Support Sulbactam-Durlobactam (SUL-DUR) Dosing Regimens in Patients with Altered Renal Function

**DOI:** 10.1093/ofid/ofad500.2166

**Published:** 2023-11-27

**Authors:** Kajal Larson, Sujata M Bhavnani, Jeffrey P Hammel, Anthony P Cammarata, John O’Donnell, Christopher M Rubino

**Affiliations:** Innoviva Specialty Therapeutics, Waltham, Massachusetts; Institute for Clinical Pharmacodynamics, Schenectady, NY; Institute for Clinical Pharmacodynamics, Schenectady, NY; Institute for Clinical Pharmacodynamics, Schenectady, NY; Entasis Therapeutics, Waltham, Massachusetts; Institute for Clinical Pharmacodynamics, Schenectady, NY

## Abstract

**Background:**

Sulbactam-durlobactam (SUL-DUR) is a β-lactam/β-lactamase inhibitor combination in development for the treatment of infections caused by *Acinetobacter baumannii*-*calcoaceticus* complex (ABC), including multidrug-resistant and carbapenem-resistant isolates. SUL-DUR is predominantly renally eliminated, with altered exposures in patients with renal impairment (RI) or with augmented renal clearance (ARC). Using population pharmacokinetic (PPK) approaches with Phase 1-3 clinical data, alternate dosing regimens to those used in the Phase 3 trial were assessed across renal function categories.

**Methods:**

A PPK model was previously constructed using data from 373 subjects, of which 110 were infected Phase 3 patients, and included the impact of statistically significant covariates, such as creatinine clearance (CL_CR_), on the variability in SUL-DUR PK. The population mean clearance of both drugs increased with increasing CL_CR_. Using this model, simulations were conducted to evaluate appropriate dosing regimens (as 3-hour intravenous infusions) for patients with RI and ARC based on PK exposure and probability of pharmacokinetic/pharmacodynamic (PK/PD) target attainment (PTA).

**Results:**

Patients with CL_CR_ < 45 mL/min had higher exposures of SUL-DUR compared to patients with CL_CR_ ≥ 60 mL/min (Table 1), suggesting that further dose adjustments may be needed in these subgroups. To streamline the dosing regimens across renal function categories, a dosing regimen of 1 g/1 g SUL-DUR q4h instead of 1.5 g/1.5 g SUL-DUR q6h was also evaluated for patients with ARC. Alternate dosing regimens (Table 2) yielded Day 3 plasma exposures that were generally contained within the 5^th^ and 95^th^ percentiles of the exposure distribution of simulated patients with normal renal function (Figure 1). Based on PK/PD targets associated with a 1-log_10_ CFU reduction from baseline on Day 1, a PTA > 90% for MIC values ≤ 4 mg/L across CL_CR_ categories was achieved in both plasma and epithelial lining fluid.

**Figure:**
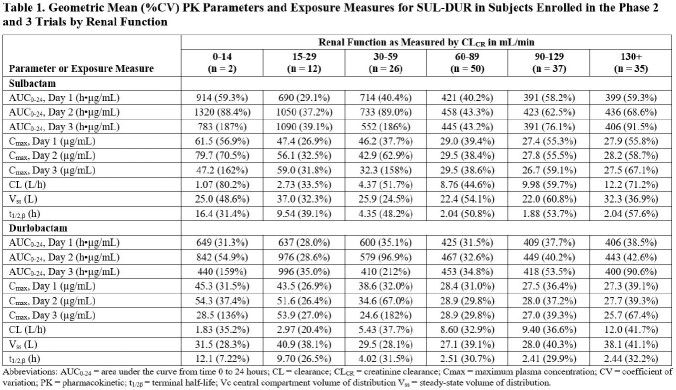

**Figure:**
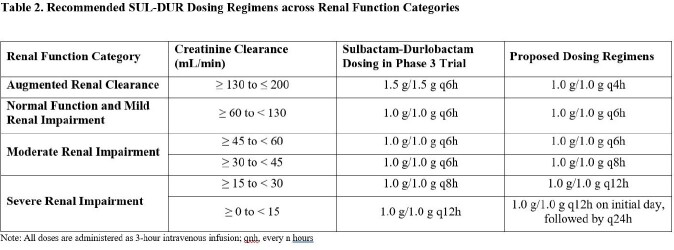

**Figure:**
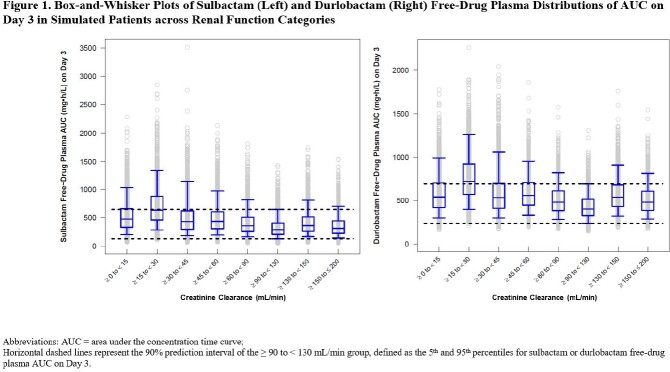

**Conclusion:**

Alternate SUL-DUR dosing regimens in simulated patients with CL_CR_ < 45 mL/min and ≥ 130 mL/min resulted in exposures that were comparable to those in patients with normal renal function with a high PTA, indicating that these doses are expected to be safe and efficacious.

**Disclosures:**

**Kajal Larson, PhD**, Entasis Therapeutics, a wholly-owned subsidiary of Innoviva: Employee|Entasis Therapeutics, a wholly-owned subsidiary of Innoviva: Stocks/Bonds **Sujata M. Bhavnani, PharmD; MS; FIDSA**, Adagio Therapeutics, Inc.: Grant/Research Support|Albany Medical Center: Grant/Research Support|Amplyx Pharmaceuticals, Inc.: Grant/Research Support|AN2 Therapeutics: Grant/Research Support|Antabio SAS: Grant/Research Support|Arcutis Biotherapeutics, Inc.: Grant/Research Support|B. Braun Medical Inc.: Grant/Research Support|Basilea Pharmaceutica: Grant/Research Support|BioFire Diagnostics LLC: Grant/Research Support|Boston Pharmaceuticals: Grant/Research Support|Cidara Therapeutics Inc.: Grant/Research Support|Cipla USA: Grant/Research Support|Crestone Inc.: Grant/Research Support|CXC: Grant/Research Support|Debiopharm International SA: Grant/Research Support|Entasis Therapeutics: Grant/Research Support|Genentech: Grant/Research Support|GlaxoSmithKline: Grant/Research Support|Hoffmann-La Roche: Grant/Research Support|ICPD: Ownership Interest|Inotrem: Grant/Research Support|Insmed Inc.: Grant/Research Support|Iterum Therapeutics Limited: Grant/Research Support|Kaizen Bioscience, Co.: Grant/Research Support|KBP Biosciences USA: Grant/Research Support|Matinas Biopharma: Grant/Research Support|Meiji Seika Pharma Co., Ltd.: Grant/Research Support|Melinta Therapeutics: Grant/Research Support|Menarini Ricerche S.p.A.: Grant/Research Support|Mutabilis: Grant/Research Support|Nabriva Therapeutics AG: Grant/Research Support|Paratek Pharmaceuticals, Inc.: Grant/Research Support|Qpex Biopharma: Grant/Research Support|Sfunga Therapeutics: Grant/Research Support|Spero Therapeutics: Grant/Research Support|Suzhou Sinovent Pharmaceuticals Co.: Grant/Research Support|Theravance: Grant/Research Support|tranScrip Partners: Grant/Research Support|University of Wisconsin: Grant/Research Support|Utility Therapeutics: Grant/Research Support|ValanBio Therapeutics Inc.: Grant/Research Support|VenatoRx: Grant/Research Support **Jeffrey P. Hammel, MS**, Adagio Therapeutics, Inc.: Grant/Research Support|Albany Medical Center: Grant/Research Support|Amplyx Pharmaceuticals, Inc.: Grant/Research Support|AN2 Therapeutics: Grant/Research Support|Antabio SAS: Grant/Research Support|Arcutis Biotherapeutics, Inc.: Grant/Research Support|B. Braun Medical Inc.: Grant/Research Support|Basilea Pharmaceutica: Grant/Research Support|BioFire Diagnostics LLC: Grant/Research Support|Boston Pharmaceuticals: Grant/Research Support|Cidara Therapeutics Inc.: Grant/Research Support|Cipla USA: Grant/Research Support|Crestone Inc.: Grant/Research Support|CXC: Grant/Research Support|Debiopharm International SA: Grant/Research Support|Entasis Therapeutics: Grant/Research Support|Genentech: Grant/Research Support|GlaxoSmithKline: Grant/Research Support|Hoffmann-La Roche: Grant/Research Support|ICPD: Employee|Inotrem: Grant/Research Support|Insmed Inc.: Grant/Research Support|Iterum Therapeutics Limited: Grant/Research Support|Kaizen Bioscience, Co.: Grant/Research Support|KBP Biosciences USA: Grant/Research Support|Matinas Biopharma: Grant/Research Support|Meiji Seika Pharma Co., Ltd.: Grant/Research Support|Melinta Therapeutics: Grant/Research Support|Menarini Ricerche S.p.A.: Grant/Research Support|Mutabilis: Grant/Research Support|Nabriva Therapeutics AG: Grant/Research Support|Paratek Pharmaceuticals, Inc.: Grant/Research Support|Qpex Biopharma: Grant/Research Support|Sfunga Therapeutics: Grant/Research Support|Spero Therapeutics: Grant/Research Support|Suzhou Sinovent Pharmaceuticals Co.: Grant/Research Support|Theravance: Grant/Research Support|tranScrip Partners: Grant/Research Support|University of Wisconsin: Grant/Research Support|Utility Therapeutics: Grant/Research Support|ValanBio Therapeutics Inc.: Grant/Research Support|VenatoRx: Grant/Research Support **Anthony P. Cammarata, M.S.**, Adagio Therapeutics, Inc.: Grant/Research Support|Albany Medical Center: Grant/Research Support|Amplyx Pharmaceuticals, Inc.: Grant/Research Support|AN2 Therapeutics: Grant/Research Support|Antabio SAS: Grant/Research Support|Arcutis Biotherapeutics, Inc.: Grant/Research Support|B. Braun Medical Inc.: Grant/Research Support|Basilea Pharmaceutica: Grant/Research Support|BioFire Diagnostics LLC: Grant/Research Support|Boston Pharmaceuticals: Grant/Research Support|Cidara Therapeutics Inc.: Grant/Research Support|Cipla USA: Grant/Research Support|Crestone Inc.: Grant/Research Support|CXC: Grant/Research Support|Debiopharm International SA: Grant/Research Support|Entasis Therapeutics: Grant/Research Support|Genentech: Grant/Research Support|GlaxoSmithKline: Grant/Research Support|Hoffmann-La Roche: Grant/Research Support|ICPD: Employee|Inotrem: Grant/Research Support|Insmed Inc.: Grant/Research Support|Iterum Therapeutics Limited: Grant/Research Support|Kaizen Bioscience, Co.: Grant/Research Support|KBP Biosciences USA: Grant/Research Support|Matinas Biopharma: Grant/Research Support|Meiji Seika Pharma Co., Ltd.: Grant/Research Support|Melinta Therapeutics: Grant/Research Support|Menarini Ricerche S.p.A.: Grant/Research Support|Mutabilis: Grant/Research Support|Nabriva Therapeutics AG: Grant/Research Support|Paratek Pharmaceuticals, Inc.: Grant/Research Support|Qpex Biopharma: Grant/Research Support|Sfunga Therapeutics: Grant/Research Support|Spero Therapeutics: Grant/Research Support|Suzhou Sinovent Pharmaceuticals Co.: Grant/Research Support|Theravance: Grant/Research Support|tranScrip Partners: Grant/Research Support|University of Wisconsin: Grant/Research Support|Utility Therapeutics: Grant/Research Support|ValanBio Therapeutics Inc.: Grant/Research Support|VenatoRx: Grant/Research Support **John O'Donnell, B.S**, Entasis Therapeutics, a wholly-owned subsidiary of Innoviva: Employee|Entasis Therapeutics, a wholly-owned subsidiary of Innoviva: Stocks/Bonds **Christopher M. Rubino, PharmD**, Adagio Therapeutics, Inc.: Grant/Research Support|Albany Medical Center: Grant/Research Support|Amplyx Pharmaceuticals, Inc.: Grant/Research Support|AN2 Therapeutics: Grant/Research Support|Antabio SAS: Grant/Research Support|Arcutis Biotherapeutics, Inc.: Grant/Research Support|B. Braun Medical Inc.: Grant/Research Support|Basilea Pharmaceutica: Grant/Research Support|BioFire Diagnostics LLC: Grant/Research Support|Boston Pharmaceuticals: Grant/Research Support|Cidara Therapeutics Inc.: Grant/Research Support|Cipla USA: Grant/Research Support|Crestone Inc.: Grant/Research Support|CXC: Grant/Research Support|Debiopharm International SA: Grant/Research Support|Entasis Therapeutics: Grant/Research Support|Genentech: Grant/Research Support|GlaxoSmithKline: Grant/Research Support|Hoffmann-La Roche: Grant/Research Support|ICPD: Ownership Interest|Inotrem: Grant/Research Support|Insmed Inc.: Grant/Research Support|Iterum Therapeutics Limited: Grant/Research Support|Kaizen Bioscience, Co.: Grant/Research Support|KBP Biosciences USA: Grant/Research Support|Matinas Biopharma: Grant/Research Support|Meiji Seika Pharma Co., Ltd.: Grant/Research Support|Melinta Therapeutics: Grant/Research Support|Menarini Ricerche S.p.A.: Grant/Research Support|Mutabilis: Grant/Research Support|Nabriva Therapeutics AG: Grant/Research Support|Paratek Pharmaceuticals, Inc.: Grant/Research Support|Qpex Biopharma: Grant/Research Support|Sfunga Therapeutics: Grant/Research Support|Spero Therapeutics: Grant/Research Support|Suzhou Sinovent Pharmaceuticals Co.: Grant/Research Support|Theravance: Grant/Research Support|tranScrip Partners: Grant/Research Support|University of Wisconsin: Grant/Research Support|Utility Therapeutics: Grant/Research Support|ValanBio Therapeutics Inc.: Grant/Research Support|VenatoRx: Grant/Research Support

